# semPower: General power analysis for structural equation models

**DOI:** 10.3758/s13428-023-02254-7

**Published:** 2023-11-10

**Authors:** Morten Moshagen, Martina Bader

**Affiliations:** Psychological Research Methods, Institute of Psychology and Education, Ulm University, Albert-Einstein-Allee 4, 89081 Ulm, Germany

**Keywords:** Confirmatory factor analysis, Model evaluation, Sample size planning, Statistical power, Structural equation modeling

## Abstract

Structural equation modeling (SEM) is a widespread and commonly used approach to test substantive hypotheses in the social and behavioral sciences. When performing hypothesis tests, it is vital to rely on a sufficiently large sample size to achieve an adequate degree of statistical power to detect the hypothesized effect. However, applications of SEM rarely consider statistical power in informing sample size considerations or determine the statistical power for the focal hypothesis tests performed. One reason is the difficulty in translating substantive hypotheses into specific effect size values required to perform power analyses, as well as the lack of user-friendly software to automate this process. The present paper presents the second version of the R package semPower which includes comprehensive functionality for various types of power analyses in SEM. Specifically, semPower 2 allows one to perform both analytical and simulated a priori, post hoc, and compromise power analysis for structural equation models with or without latent variables, and also supports multigroup settings and provides user-friendly convenience functions for many common model types (e.g., standard confirmatory factor analysis [CFA] models, regression models, autoregressive moving average [ARMA] models, cross-lagged panel models) to simplify power analyses when a model-based definition of the effect in terms of model parameters is desired.

Structural equation modeling (SEM) is a commonly employed statistical technique to test various types of substantive hypotheses. These hypotheses may refer to global aspects of a model, such as whether the assumed structure provides an adequate description of the population mechanics, but also to more local aspects, such as whether a particular model parameter differs from zero, across groups, or across measurement occasions. Regardless of the particular hypothesis under scrutiny, it is vital to rely on a sufficiently large sample size to achieve an adequate degree of statistical power to detect the hypothesized effect.

Statistical power is a concept arising in the context of classical (frequentist) null-hypothesis significance testing, where a null hypothesis (H0) is evaluated against an alternative hypothesis (H1). In any hypothesis test, two types of decision errors may occur: the alpha-error of incorrectly rejecting a true null hypothesis (and thus incorrectly accepting a false alternative hypothesis) and the beta-error of incorrectly retaining a false null hypothesis (and thus incorrectly rejecting a true alternative hypothesis). Statistical power is the complement of the beta-error and gives the probability of rejecting a null hypothesis if this hypothesis is factually wrong (and thus to correctly accept a true alternative hypothesis).

As an example, consider a simple confirmatory factor analysis (CFA) model involving two factors that are measured by three indicators each, and assume that the interest lies in the correlation between the factors. To test the null hypothesis that the factors are uncorrelated versus the alternative hypothesis that the correlation differs from zero, one can compare a model that freely estimates the factor correlation (reflecting the H1) with a model that restricts the correlation between these factors to zero but is otherwise identical (reflecting the H0). If the restricted model fits the data significantly worse than the unrestricted model, the null hypothesis of a zero correlation between the factors is rejected, in turn lending support for the alternative hypothesis that the correlation between the factors differs from zero. Statistical power now gives the probability that the outcome of the statistical test contrasting the H0 and the H1 turns out significant on a certain alpha-error level with a certain sample size and given a certain discrepancy between the H0 and the H1 in the population. For instance, suppose that the true correlation between the factors in the population is *r* = .20 and that all nonzero primary loadings equal .50. Then, the statistical power to reject the factually incorrect null hypothesis (of *r* = 0) given alpha = .05 and a sample size of *N* = 125 is just 20%. Stated differently, in four out of five samples (each of size *N* = 125) drawn randomly from the population, one will not detect that the factors are in fact correlated. Indeed, to obtain a more reasonable power of 80% in this scenario, a sample size of *N* = 783 is required.

As is evident from this example, hypotheses tests are only meaningful to the extent that statistical power is sufficiently high, because otherwise a nonsignificant test outcome provides little information regarding the veracity of the tested hypothesis. Conversely, even miniscule discrepancies between the H0 and the H1 that are of no practical relevance may turn out significant when statistical power is high. Statistical power is thus an integral part of sample size planning and statistical hypothesis testing more generally.

Unfortunately, statistical power analyses are often not reported at all in applications of SEM (Jackson et al., 2009), or are only performed with respect to a single hypothesis. However, power is always tied to a particular hypothesis and might vary strongly depending on which hypothesis is considered. For instance, in a simple two-wave cross-lagged panel model (CLPM; e.g., Newsom, 2015) involving four factors, each comprising three indicators loading by .50, *N* = 252 observations are required to yield 80% power on alpha = .05 to detect global misspecifications corresponding to a root-mean-square error of approximation (RMSEA) ≥ .05. In contrast, *N* = 4629 observations are required to detect a cross-lagged effect ≥ .10 in the same model with an equal level of power. Correspondingly, power analyses should be performed for the focal hypothesis of interest, which is rarely done in practice. One reason for the notable absence of power considerations is arguably the lack of powerful software tools easily allowing for the assessment of power for both global and local hypotheses. The present paper therefore introduces the *semPower* 2 package for the R environment, which supports various types of and approaches to power analyses covering both model-free and model-based definitions of the hypothesis of interest. In the remainder of this paper, we first provide the technical background underlying power analysis, and then illustrate how to use semPower 2 based on illustrative examples.

## Statistical background

Statistical power depends on (a) the degree to which the null hypothesis is wrong (the magnitude of effect), (b) the sample size (*N*), (c) the specified alpha-error, and (d) the degrees of freedom (*df*). Everything else being equal, power will be higher for a larger effect, a larger sample size, a higher alpha-error, and fewer *df*. In a power analysis, one of these quantities is computed as a function of the other quantities, giving rise to different types of power analyses (Faul et al., 2007). *A priori power analyses* determine the required number of observations to detect a certain effect with a desired power, given alpha and *df*, and are thus performed prior to data collection to inform sample size planning. *Post hoc power analyses* determine the achieved power to detect a certain effect with a given sample size, alpha, and *df*, and are thus performed after data collection to judge whether the given sample size yields a power that is sufficiently high for a meaningful test of a certain hypothesis. *Compromise power analyses* determine the alpha- and the beta-error, given the ratio between alpha and beta, the sample size, the effect, and the *df*, and are used to determine which decision rule to apply when the error probabilities should meet a desired ratio, such as being equal (see Moshagen & Erdfelder, 2016, for details).

To understand how a power analysis can be performed, a brief discussion of model testing is required. Generally, when testing a model representing the H0 against a more general alternative model representing the H1, a likelihood-ratio test (LRT) can be performed:

1 $$LRT = -2 ln\left(\frac{{L}_{\mathrm{M}0}}{{L}_{\mathrm{M}1}}\right)$$

2 $$=\left(N-1\right){(\widehat{F}}_{\mathrm{M}0}-{\widehat{F}}_{\mathrm{M}1})$$

3 $$= {\upchi }_{\mathrm{M}0}^{2} - {\upchi }_{\mathrm{M}1}^{2}$$

*L*_M0_ is the likelihood of the hypothesized (H0) model and *L*_M1_ is the likelihood of a more general (H1) model, where the H0 model must be statistically nested in the H1 model (i.e., the parameter space of the H0 model must be a subset of the parameter space of the H1 model; Bentler & Satorra, 2010). In SEM, maximizing the likelihood is equivalent to minimizing the maximum likelihood (ML) fitting function (Jöreskog, 1967), so the LRT can also be obtained by considering the sample-size-weighted difference in the minimized sample values of the fitting function for the H0 model (${\widehat{F}}_{\mathrm{M}0}$) and the H1 model (${\widehat{F}}_{\mathrm{M}1}$).

If the H0 model is correct, assuming multivariate normality, a sufficiently large sample size, and several mild regularity conditions (Browne, 1984; Yuan & Bentler, 2007), the LRT asymptotically follows a central χ^2^(*df*) distribution with *df* = *df*_M1_ − *df*_M0_, so it can be computed in practice as the difference between the ${\upchi }^{2}$-model test statistics associated with the H0 (${\upchi }_{\mathrm{M}0}^{2})$ and the H1 model (${\upchi }_{\mathrm{M}1}^{2}$). If the H0 model is invalid, however, the LRT—again assuming multivariate normality, a sufficiently large sample size, and several regularity conditions^1^ (Satorra & Saris, 1985; Steiger et al., 1985)—asymptotically follows a noncentral χ^2^(*df*, λ) distribution with non-centrality parameter λ and expected value *df* + λ, so that λ shifts the noncentral χ^2^(*df*, λ) distribution to the right of the central ${\upchi }^{2}$ distribution. The non-centrality parameter is the sample-size-weighted difference in the population minima, *F*_0_, of the fitting function, $\uplambda =\left(N-1\right){(F}_{0,\mathrm{M}0}-{F}_{0,\mathrm{M}1})$. Note that the minimized sample value of the fitting function, $\widehat{F}$, is a biased estimate of *F*_0_ (McDonald, 1989) with an expected value of *F*_0_ + *df* / (*N* − 1). The difference in the population minima between the H0 and the H1 expresses the extent to which the H0 is incorrect (with respect to the H1) and thereby defines the magnitude of effect.

As is evident from Eq. 1, an LRT always involves a comparison of the hypothesized H0 model against an alternative H1 model. The latter can either be explicitly specified or refer to the saturated model. Generally, a structural equation model in which the number of free parameters is identical to the number of observed means, variances, and covariances is saturated, so the saturated model typically just estimates each observed mean, variance, and covariance without any constraint. Consequently, a saturated H1 model can always describe the observed data perfectly and shows ${F}_{0,\mathrm{M}1}$= 0 on zero *df*.

Returning to the introductory example concerning the correlation between two factors in a CFA model, the H0 model restricting the correlation to zero could thus be compared either against a less restricted model that freely estimates the correlation, but is otherwise identical to the H0 model (leading to *df* = 1), or against the saturated model freely estimating all variances and covariances (leading to *df* = 9). Although either approach is valid, the comparison model should generally be chosen such that it allows for an immediate test of the hypothesis articulated in the H0 model (Jöreskog, 1978). Thus, when a (local) hypothesis explicitly refers to a zero correlation between two factors, the suitable comparison model should only differ in this particular parameter. However, when a (global) hypothesis states that the model as a whole adequately describes the data, the saturated model is the proper comparison model.

**Fig. 1:**
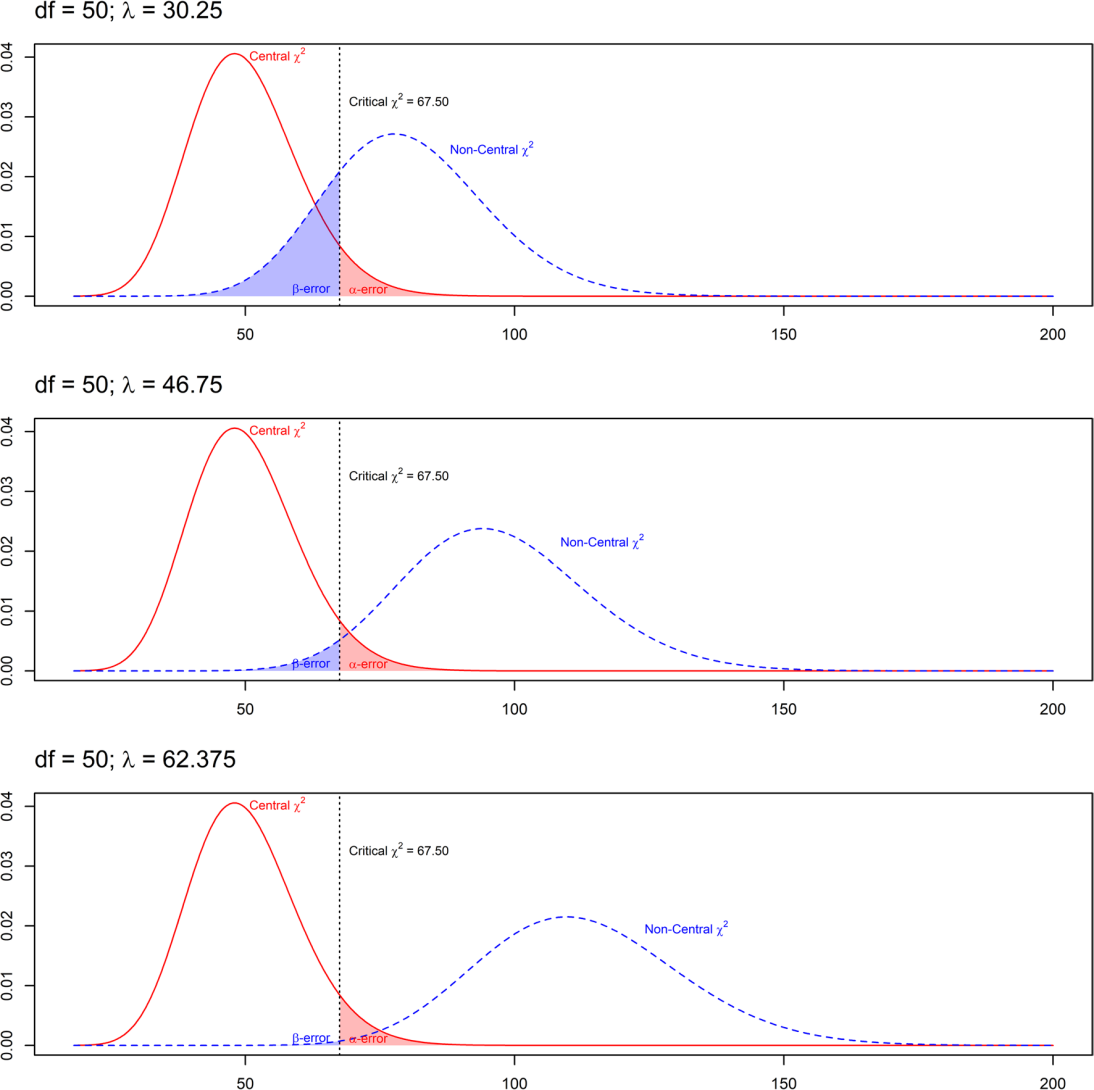
Central and noncentral χ^2^ distributions, critical χ^2^ value, and associated decision errors. *Note.* Central χ^2^(*df*) distribution (solid line) and noncentral χ^2^(*df*, λ) distributions (dashed lines) with *df* = 50 and (from top to bottom) non-centrality parameters of λ = 30.25, λ = 46.75, and λ = 62.375. The dotted vertical line indicates a critical value for ${\upchi }_{\mathrm{c}}^{2}$ of 67.50, which corresponds to alpha = .05. The beta-errors are β = .20, β = .03, and β = .003, respectively

Figure 1 exemplarily illustrates how the alpha- and beta-error arise in a hypothesis testing situation involving 50 *df* and three different values for the non-centrality parameter. The null hypothesis is rejected when the *p*-value associated with the empirically observed LRT falls below the alpha-error. This is equivalent to evaluating whether the value of the LRT exceeds the critical ${\upchi }_{\mathrm{c}}^{2}$-value corresponding to the chosen alpha-error (e.g., ${\upchi }_{\mathrm{c}}^{2}(df=50)=67.50$ for alpha = .05). Note that neither the alpha-error nor the implied critical ${\upchi }_{\mathrm{c}}^{2}$-value change as a function of the non-centrality parameter. The area of the central χ^2^(*df*) distribution to the right of the critical value corresponds to the alpha-error, whereas the beta-error is the area of the noncentral χ^2^(*df*, λ) distribution to the left of the critical value, i.e.,

4 $$\beta ={\int }_{0}^{{\upchi }_{\mathrm{c}}^{2}}{f}_{{\upchi }^{2}(df,\uplambda )}(x) dx$$

Correspondingly, the beta-error (and thus statistical power, 1 − β) can be computed given the non-centrality parameter, which depends on *N* and the magnitude of effect, and the critical value, which depends on the *df* and the alpha-error. Choosing a smaller alpha-error increases the beta-error by increasing the implied critical value ${\upchi }_{\mathrm{c}}^{2}$. The beta-error (but not the alpha-error) is also a function of the overlap between the central χ^2^(*df*) and the noncentral distributions χ^2^(*df*, λ), which decreases with increasing non-centrality parameter λ, and thus with increasing sample size and with an increasing effect.

## Analytical versus simulated power analysis

Power analyses can be performed either analytically drawing on asymptotic theory or based on a simulation approach. In SEM, analytical power analyses as described above require the definition of the effect of interest in terms of a measure that shows a direct relation to the non-centrality parameter, which is afforded by all non-centrality-based measures of fit such as the minimum of the employed fitting function itself or derived indices such as the RMSEA (because ${F}_{0}=df \cdot {RMSEA}^{2}$; e.g., MacCallum et al., 1996). Analytical power analyses are thus inherently model-free in the sense that the achieved power to reject a H0 model exhibiting a specific degree of misspecification on a certain *df* and a defined alpha-error with a certain *N* is always the same, regardless of whether the model is a CFA model, a CLPM, a multigroup model, or any other SEM model. Beyond allowing for the direct comparison across different model types, reliance on a non-centrality-based effect size for power analyses is particularly useful when only the overall degree of misfit of a particular model is of interest, or when the effect of interest spreads across many parameters (such as in tests of measurement invariance or when testing a bifactor versus a higher-order structure). However, a common problem is that a focal hypothesis often refers to a specific model parameter (such as a hypothesized cross-lagged effect in a CLPM of a certain magnitude), but it is not straightforward to relate such hypotheses to a particular value of a non-centrality-based effect size.

Unlike analytical power analyses, simulated power analyses perform a simulation study to obtain an empirical estimate of power. Specifically, a model is defined that describes the true state of affairs in the population (in terms of the number of observed variables, the number of latent factors, the magnitude of loadings, (residual-)variances, covariances between latent variables, etc.), which in turn defines the population variance–covariance matrix and population means (if these are part of the model). Based on these population characteristics, random data sets are repeatedly generated and analyzed (e.g., 1000 times). In addition, an analysis model is defined, which implements at least one restriction that is factually wrong (representing the H0). When the H1 does not refer to the saturated model, a more general, explicit H1 comparison model can also be defined. Then, an empirical estimate of power is obtained by the proportion of significant outcomes of the LRT on a given alpha-error level.

As a consequence, simulated power analyses are directly tied to a particular model representing the H0 and define the effect of interest in terms of the difference in a particular (set of) parameter(s) that are embedded in a certain model structure with particular population values for all parameters (Satorra & Saris, 1983; Wang & Rhemtulla, 2021). Of note, the actual effect in terms of *F*_0_ not only is a function of the parameters that differ between the H0 and the H1 model, but also depends on all other parameters in the model (e.g., Moshagen & Auerswald, 2018; Wang & Rhemtulla, 2021). Therefore, the level of statistical power is generally specific to the particular model under scrutiny and might change not only with the population difference of a particular parameter, but also as a function of the value of other parameters.

However, although the model-free versus model-based definition of the effect of interest is indeed a key difference between analytical and simulated power analysis, analytical power analyses can also be performed in a model-based manner. To this end, analytical power analysis requires that an effect defined in terms of model parameters is transformed into an effect based on non-centrality (Saris & Satorra, 1993). This can be achieved by evaluating the discrepancy between the population variance–covariance matrix (${\boldsymbol{\Sigma}}$) and the model-implied variance–covariance matrix ($\widehat{{\boldsymbol{\Sigma}}}$) (and mean vectors ${\boldsymbol{\upmu}}$ and $\widehat{{\boldsymbol{\upmu}}}$, if means are part of model) as measured via a suitable fitting function, such as weighted least squares (Browne, 1984) or ML. By default, semPower 2 relies on the ML fitting function assuming continuously distributed data:

5 $${F}_{0}\left({\boldsymbol{\Sigma}},\widehat{{\boldsymbol{\Sigma}}}\right)={ln}\left|\widehat{{\boldsymbol{\Sigma}}}\right|-{ln}\left|{\boldsymbol{\Sigma}}\right|+tr({\boldsymbol{\Sigma}}{\widehat{{\boldsymbol{\Sigma}}}}^{-1})-p + ({\boldsymbol{\mu}}-\widehat{{\boldsymbol{\mu}}}){\widehat{{\boldsymbol{\Sigma}}}}^{-1}({\boldsymbol{\mu}}-\widehat{{\boldsymbol{\mu}}})$$

In more practical terms, the model representing the H0 is fitted to a population covariance matrix ${\boldsymbol{\Sigma}}$ and the population means ${\boldsymbol{\upmu}}$. The resulting parameter estimates are used to construct the model-implied covariance matrix $\widehat{{\boldsymbol{\Sigma}}}$ and means $\widehat{{\boldsymbol{\upmu}}}$ associated with the H0. Finally, Eq. 5 is used to obtain the population minimum of the ML fitting function *F*_0_. Effectively, a model-based power analysis can be transformed into a model-free power analysis using *F*_0_ as an effect size metric. In this way, analytical power can always be performed for both model-based and model-free definitions of the effect. In contrast, simulated power analyses are necessarily model-based.

Beyond their greater versatility in terms of the definition of the effect of interest, analytical power analyses are generally preferable over simulated power analyses to the extent that the assumptions underlying the asymptotically expected χ^2^(*df*) distributions under the null and the alternative hypothesis are met. This is because computation is much faster (instantly); in particular, when an a priori power analysis is performed, there is no random error resulting from the sampling process, and there are no additional intricacies associated with model estimation, such as non-convergence. However, when the assumptions underlying analytical power analysis are violated, simulated power analysis may offer a more realistic assessment, provided that these violations are embedded in the simulation and that the defined population structure and data-generating process can be considered representative of empirical reality. Simulated power analysis further offers the advantage that it may provide information beyond statistical power, such as rates of proper convergence and parameter recovery, which helps to inform sample size requirements more generally. In sum, both analytical and simulated power analyses have their advantages and are best used complementarily.

## Software for power analysis in SEM

Despite the importance of statistical power in sample size planning and hypothesis testing, available software programs and packages directed towards this goal are subject to various shortcomings. To our knowledge, existing tools rely on either an analytical or a simulated approach to power analysis, but do not provide both options. General power analysis software programs such as G*Power (Faul et al., 2007) do not offer effect sizes relevant for SEM. Existing tools providing analytical power analyses specifically for SEM (e.g., MacCallum et al., 1996, 2006; Preacher & Coffman, 2006) require provision of a non-centrality-based measure of effect such as the RMSEA and are thus limited to a model-free approach. Although simulation approaches to statistical power necessarily allow for a model-based definition of the effect of interest, most existing tools implement post hoc power analyses only and are typically limited to particular model types (Kievit et al., 2018; Mulder, 2022; Schoemann et al., 2017; Zhang & Liu, 2018). A number of options providing either analytical (Jak et al., 2021; Miles, 2003; Z. Zhang & Yuan, 2018) or simulated power analysis (Muthén & Muthén, 2002; Wang & Rhemtulla, 2021) allow for a generic model-based definition of the effect, but these require the manual definition of the values of all parameters of the population model. Such a manual definition is cumbersome and potentially error-prone, in particular when more complex models are considered and it is desired to define the model in terms of standardized parameters, which requires rather sophisticated knowledge concerning the proper definition of the residual variances (see e.g., Bader & Moshagen, 2022).

As a remedy, the present paper introduces the second version of the semPower package—semPower 2—for the R environment, which can be obtained from CRAN (https://cran.r-project.org/package=semPower) or from GitHub (https://github.com/moshagen/semPower), which also hosts its source code. The semPower 2 package is a massive improvement and extension over the first version of semPower (Moshagen & Erdfelder, 2016), which only provided analytical power analysis for common effect size metrics or based on a covariance matrix input (thereby offering model-based power analysis via rather cumbersome procedures; see Jobst et al., 2023). Specifically, semPower 2 provides a priori, post hoc, and compromise power analysis, both analytical and simulated, for structural equation models with or without latent variables, supports multigroup settings, and offers highly abstracted input for many common model types to simplify the model specification when a model-based definition of the effect in terms of model parameters is desired.

The remainder of this paper presents an overview of semPower 2. We illustrate various types of analytical power analyses based on both model-free and model-based definitions of the effect, and also briefly describe how simulated power analyses can be performed.

## semPower 2 overview

In the following, an introductory overview of the core features of semPower 2 is presented. First, we provide various examples of how to perform analytical model-free power analyses comparing the H0 model either against the saturated model or against a less restrictive (but not saturated) competing model. We then illustrate analytical model-based power analyses based on the convenience functions of semPower 2 using several examples. Finally, we move to simulation approaches to model-based power analysis. Readers are referred to the extensive online documentation available at https://moshagen.github.io/semPower/ for a complete and more detailed description of the capabilities of semPower 2.

## Analytical model-free power analysis

We first consider analytical model-free power analyses comparing the H0 model against the saturated H1 comparison model. Any model-free power analysis requires provision of the measure (effect.measure) and magnitude (effect) of effect that is to be detected as well as the *df*. In an a priori power analysis, the desired power also needs to be specified, so that a model-free a priori power analysis can be performed by calling the semPower function setting type = 'a-priori' and corresponding further arguments. For instance, the following code requests the required sample size to detect misspecifications of a model involving 50 *df* corresponding to RMSEA ≥ .05 on alpha = .05 with a power of 80%:
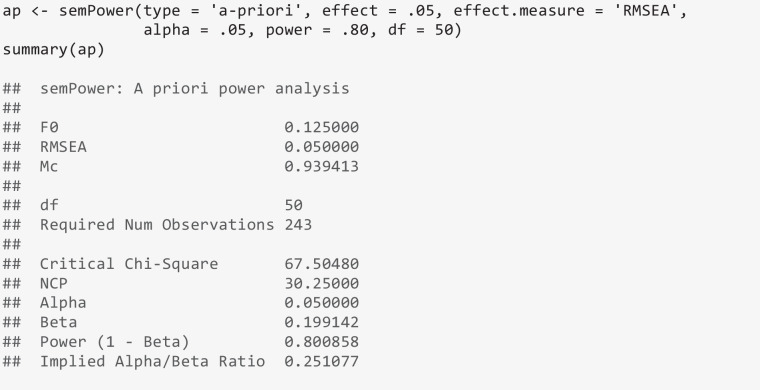


The output first provides several non-centrality measures of effect, here showing that an RMSEA of .05 (on 50 *df*) is equivalent to *F*_0_ = .125 and to McDonald’s (1989) index of non-centrality^2^ (Mc) of .939. The output further indicates a required sample size of *N* = 243 to detect this effect with a power of 80% on alpha = .05.

A post hoc power analysis computes the achieved power with a given sample size, so the semPower function with type = 'post-hoc' expects the N argument. For instance, the following code computes the achieved power with a sample size of *N* = 500 in the same situation as above:
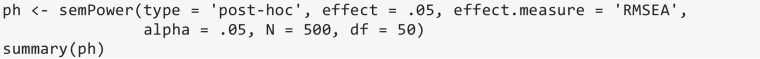


This yields an output structured identically to the one of an a priori power analysis and shows that with *N* = 500, the achieved power to detect misspecifications of a model involving 50 *df* amounting to RMSEA ≥ .05 on alpha = .05 is 99.7%. This implies that committing an alpha-error is about 15 times as likely as committing a beta-error (the ratio of alpha to beta is .05 / .00335 = 14.9).

**Fig. 2:**
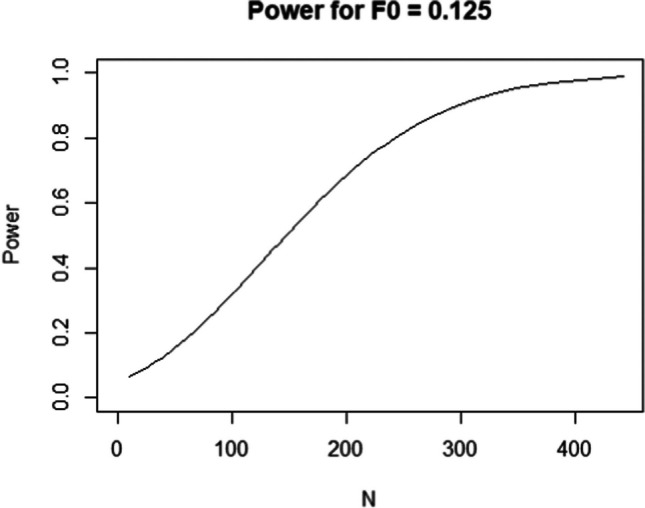
Statistical power as a function of sample size to detect RMSEA = .05 on 50 df (F_0_ = 0.125)


The result of any (model-free or model-based) a priori or post hoc power analysis can also be plugged into the semPower.powerPlot function to produce a figure showing how power for the specified effect varies as a function of sample size. Thus, a plot akin to Fig. 2 is produced by calling

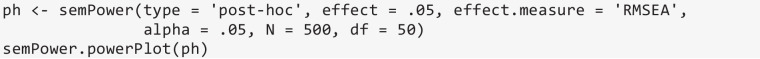


If the goal is to determine a decision rule (i.e., the critical value ${\upchi }_{\mathrm{c}}^{2}$ of the ${\upchi }^{2}$ test statistic) that balances the error probabilities, a compromise power analysis can be used. The semPower function with type = 'compromise' expects the abratio argument defining the desired ratio between alpha and beta. For instance, the following code determines ${\upchi }_{\mathrm{c}}^{2}$ and the associated error probabilities, such that the alpha- and beta-error are equal:
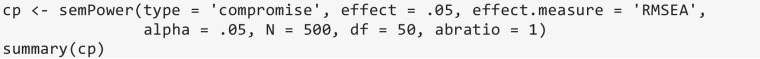


The output is again structured identically to the one shown above and indicates that choosing a critical value of ${\upchi }_{\mathrm{c}}^{2}=74.64$ is associated with equal error probabilities, alpha = beta = .013.

### Power for effect-size differences

The examples above were concerned with the situation that an H0 model is compared against a saturated H1 model. A common scenario is to test two competing models against each other, where the more restrictive H0 model (involving more *df*) is compared against a less restrictive H1 model (involving fewer *df*), so that power is to be determined concerning the difference between these models in terms of overall fit. This can be achieved by providing a vector of effect sizes to the effect argument. For instance, the following determines the required sample size to test a more general H1 model exhibiting an RMSEA of .04 on 44 *df* against a nested H0 model exhibiting an RMSEA of .05 on 41 *df *which shows that *N* = 247 observations are necessary to detect a difference of RMSEA = .01 between these models with a power of 80%.
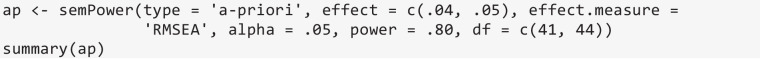


The same approach can also be used in multiple-group contexts, when sufficient power is desired to detect whether certain cross-group constraints on the model parameters (such as equal loadings across groups) are tenable. The general syntax is the same as above, but now the N argument also needs to be set, which gives the number of observations per group in compromise and post hoc power analyses and the group weights in a priori power analyses. For example, it has been suggested that a decline of ≥ .02 in the Mc associated with a metric in comparison to a configural invariance model may be considered a meaningful departure from measurement invariance (Cheung & Rensvold, 2002). Accordingly, the following asks for the required sample size to detect that a model exhibiting an Mc of .99 on 57 *df* differs from a model exhibiting an Mc of .97 on 69 *df* in a three-group model, where all groups are of equal size (N = c(1, 1, 1)):
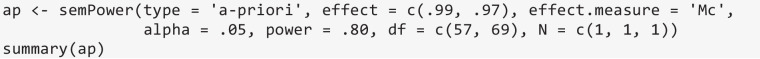


The results indicate that *N* = 426 (*n* = 142 by group) observations are required to yield a power of 80%.

## Analytical model-based power analysis

semPower 2 provides a number of convenience functions (see Table 1 for an overview) to perform (both analytical and simulated) a priori, post hoc, and compromise power analyses using a model-based definition of the effect of interest in terms of particular values for the model parameters. These convenience functions offer a high level of abstraction to simplify the definition of the model and the effect of interest. In addition, semPower 2 includes a more generic function (semPower.powerLav), which allows one to perform model-based power analyses for models and hypotheses not covered by any more specific convenience function dedicated to a particular model structure and thereby provides a high degree of flexibility.

**Table 1:** semPower 2 functions providing model-based power analyses

Function	Model	Hypotheses
semPower.powerCFA	CFA	${\Lambda }_{i,j}=0$ ${\phi }_{i,j}=0$ ${\phi }_{i,j}={\phi }_{k,l}$ ${\phi }_{i,j,m}={\phi }_{i,j,n}$
semPower.powerBifactor	Bifactor	${\phi }_{i,j}=0$ ${\phi }_{i,j}={\phi }_{k,l}$ ${\phi }_{i,j,m}={\phi }_{k,l,n}$
semPower.powerRegression	Single linear regression	${B}_{i,j}=0$ ${B}_{i,j}={B}_{i,k}$ ${B}_{i,j,m}={B}_{i,j,n}$
semPower.powerMediation	Mediation	${B}_{i,j}\cdot {B}_{k,l}=0$ ${B}_{i,j,m}\cdot {B}_{k,l,m}={B}_{i,j,n}\cdot {B}_{k,l,n}$
semPower.powerPath	General regression	${B}_{i,j}=0$ ${B}_{i,j}={B}_{k,l}$ ${B}_{i,j,m}={B}_{i,j,n}$
semPower.powerAutoreg	Autoregression	${B}_{i,j}=0$ ${B}_{i,j}={B}_{k,l}$ ${B}_{i,j,m}={B}_{i,j,n}$ ${\psi }_{i,j}=0$ ${\psi }_{i,j}={\psi }_{k,l}$ ${\psi }_{i,j,m}={\psi }_{i,j,m}$ ${\psi }_{i,j,k}={\psi }_{k,l,m}$ ${\mathrm{\alpha }}_{i}=0$ ${\mathrm{\alpha }}_{i}={\alpha }_{j}$ ${\mathrm{\alpha }}_{im}={\alpha }_{in}$
semPower.powerARMA	ARMA	${B}_{i,j}=0$ ${B}_{i,j}={B}_{k,l}$ ${B}_{i,j,m}={B}_{i,j,n}$ ${\psi }_{i,j}=0$ ${\psi }_{i,j}={\psi }_{k,l}$ ${\psi }_{i,j,\mathrm{n}}={\psi }_{i,j,m}$ ${\mathrm{\alpha }}_{i}=0$ ${\mathrm{\alpha }}_{i}={\alpha }_{j}$ ${\mathrm{\alpha }}_{im}={\alpha }_{in}$
semPower.powerCLPM	CLPM	${B}_{i,j}=0$ ${B}_{i,j}={B}_{k,l}$ ${B}_{i,j,m}={B}_{i,j,n}$ ${\psi }_{i,j}=0$ ${\psi }_{i,j}={\psi }_{k,l}$ ${\psi }_{i,j,k}={\psi }_{k,l,m}$
semPower.powerRICLPM	RI-CLPM	${B}_{i,j}=0$ ${B}_{i,j}={B}_{k,l}$ ${B}_{i,j,m}={B}_{i,j,n}$ ${\psi }_{i,j}=0$ ${\psi }_{i,j}={\psi }_{k,l}$ ${\psi }_{i,j,k}={\psi }_{i,j,m}$
semPower.powerMI	Multigroup invariance	${\Lambda }_{m}={\Lambda }_{n}$ ${\uptau }_{m}={\uptau }_{n}$, ${\Theta }_{m}={\Theta }_{n}$ ${\phi }_{m}={\phi }_{n}$ ${\mathrm{\alpha }}_{m}={\alpha }_{n}$
semPower.powerLI	Longitudinal invariance	${\Lambda }_{m}={\Lambda }_{n}$ ${\uptau }_{m}={\uptau }_{n}$, ${\Theta }_{m}={\Theta }_{n}$ ${\phi }_{m}={\phi }_{n}$ ${\mathrm{\alpha }}_{m}={\alpha }_{n}$
semPower.powerLav	Generic	Any

*Note.* CFA = confirmatory factor analysis. ARMA = autoregressive moving average. CLPM = cross-lagged panel model. RI-CLPM = random intercept cross-lagged panel model. The model matrices are defined in Eqs. 6-9. Indices *i*, *j*, *k*, *l* refer to a particular element of a given vector or matrix; indices *m* and *n* denote different groups.

To perform analytical model-based power analyses, a population model and a corresponding analysis model are transformed into the population and model-implied covariance matrices (and mean vectors if these are part of the model), assuming continuously distributed data. In the context of CFA models, the population variance–covariance matrix (${\boldsymbol{\Sigma}}$) is given by

6 $${\boldsymbol{\Sigma}}={\boldsymbol{\Lambda}}{\boldsymbol{\Phi}}{{\boldsymbol{\Lambda}}}^{\boldsymbol{^{\prime}}}+{\boldsymbol{\Theta}},$$

where ${\boldsymbol{\Lambda}}$ is the loading matrix, ${\boldsymbol{\Phi}}$ is the variance–covariance matrix of the factors, and ${\boldsymbol{\Theta}}$ is the variance–covariance matrix of the indicators. The population means (${\boldsymbol{\mu}}$) are

7 $${\boldsymbol{\mu}}={\boldsymbol{\tau}}+{\boldsymbol{\Lambda}}{\boldsymbol{\alpha}}$$

where ${\boldsymbol{\tau}}$ is a vector of indicator intercepts and ${\boldsymbol{\alpha}}$ denotes a vector containing the latent factor means. In more general SEM models, ${\boldsymbol{\Sigma}}$ can be computed by

8 $${\boldsymbol{\Sigma}}={\boldsymbol{\Lambda}}{(\boldsymbol{I}-\boldsymbol{B})}^{-1}{\boldsymbol{\Psi}}{\left[{\left(\boldsymbol{I}-\boldsymbol{B}\right)}^{-1}\right]}^{\boldsymbol{^{\prime}}}{{\boldsymbol{\Lambda}}}^{\boldsymbol{^{\prime}}}+{\boldsymbol{\Theta}},$$

where **B** contains the regression coefficients between the factors and ${\boldsymbol{\Psi}}$ is the (residual) variance–covariance matrix between the latent factors. The means are then given by

9 $${\boldsymbol{\mu}}={\boldsymbol{\tau}}\boldsymbol{ }+{\boldsymbol{\Lambda}}{\left(\boldsymbol{I}-\boldsymbol{B}\right)}^{-1}{\boldsymbol{\alpha}}.$$

In any power analysis, the relevant model matrices are defined first, so that ${\boldsymbol{\Sigma}}$ and ${\boldsymbol{\mu}}$ in the population can be obtained. In analytical model-based power analyses, the model implementing the null hypothesis of interest is then fitted to the population variance–covariance matrix using the lavaan package (Rosseel, 2012), so that the population minimum *F*_0_ along with the resulting model-implied variance covariance matrix $(\widehat{{\boldsymbol{\Sigma}}})$ and mean vector ($\widehat{{\boldsymbol{\mu}}})$ are obtained based on the estimated parameters. In simulated power analyses, the analysis model is fitted repeatedly to random data generated from a population with ${\boldsymbol{\Sigma}}$ and ${\boldsymbol{\mu}}$.

Regardless of which particular function is used to perform a model-based power analysis, a number of decisions always needs to be made. First, and obviously, the type of power analysis needs to be defined. Second, one needs to set the proper comparison model via the comparison argument. This can be either the saturated model (comparison = 'saturated') or a less restricted model that merely differs from the analysis model in the absence of the constraint(s) defining the null hypothesis (comparison = 'restricted'). Finally, the measurement model for the factors (i.e., ${\boldsymbol{\Lambda}}$) needs to be defined. semPower 2 provides three ways to do this: by providing the complete loadings matrix via the Lambda argument, by providing the nonzero primary loadings on each factor via the loadings argument, or by providing a single loading magnitude to apply to each indicator of a factor or to all indicators (loadM) along with the number of indicators by factor (nIndicators). Given any definition of ${\boldsymbol{\Lambda}}$, semPower 2 defines the variance–covariance matrix of the residuals ${\boldsymbol{\Theta}}$ as a diagonal matrix with elements such that the variances of the indicators are equal to 1, implying that the loadings are in a standardized metric.

In what follows, power analyses for hypotheses arising in a CFA model, a CLPM model, and a generic path model are described to provide illustrative examples. In all cases, an a priori power analysis (type = 'a-priori') to yield a power of 80% (power = .80) on alpha = .05 (alpha = .05) in reference to a less restricted alternative model (comparison = 'restricted') is requested.

### Power analysis for hypotheses arising in a CFA model

semPower 2 provides prebuilt methods to perform power analyses concerning the following hypotheses arising in a standard CFA model: (a) detect that a loading differs from zero, (b) detect that a correlation differs from zero, (c) detect that a correlation differs across groups, and (d) detect that two correlations differ from each other. Here we restrict ourselves to examples covering the latter three hypotheses.

Consider the introductory example of a CFA model involving two factors that are measured by three indicators each, and suppose one is interested in the sample size required to detect that the correlation between the factors is *r* ≥ .20. This type of hypothesis is set via the nullEffect = 'cor = 0' argument. The Phi argument defines the correlation between the factors in the population to be .20. The measurement model is defined using the loadings argument, which is a list of vectors giving the (nonzero primary) loadings on each factor. In line with the introductory example, assume that all loadings are equal to .50. Running semPower.powerCFA with these arguments provides an output structured identically to the one of a corresponding model-free power analysis and indicates that 783 observations are required to detect this effect with a power of 80% on alpha = .05.
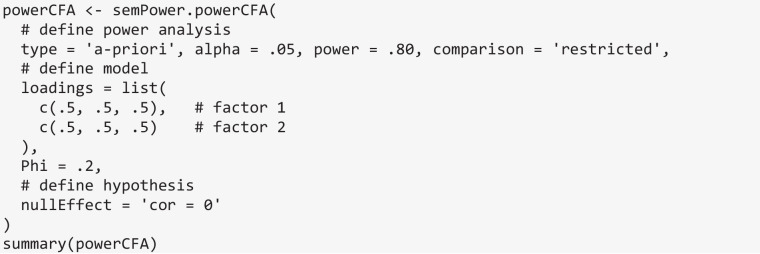


The results variable (powerCFA) also contains additional information such as the population and model-implied variance–covariance matrices (Sigma, SigmaHat) and means (mu, muHat; if these are part of the model) as well as lavaan model strings representing the H0 (modelH0) and the H1 (modelH1) models. These model strings can be plugged into lavaan to verify that the population was defined as intended:
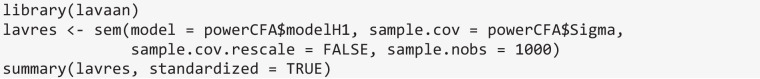


It should be noted that the actual degree of discrepancy between the H0 and the H1 models (in terms of *F*_0_) depends not only on the magnitude of the population correlation between the factors, but also on other parameters in the model. Indeed, power generally varies strongly with the number of indicators and the loading magnitude. When repeating the analysis above assuming five indicators for each factor with loadings of (.70, .80, .90, .70, .90) and (.80, .60, .90, .50, .70), respectively, the required *N* drops to 237. It is crucial to take care in defining appropriate factor loadings, which should be guided by previous empirical results.

semPower.powerCFA can also be used to perform power analyses regarding whether a correlation between factors differs across two or more groups by setting nullEffect = 'corA = corB'. Generally, semPower 2 uses the “A” / “B” suffix (e.g., corA = corB) to refer to equality constraints across multiple groups, whereas the “X” / “Z” suffix (e.g., corX = corZ) is used to refer to equality constraints across different parameters in a single group (see below). The syntax for power analyses in a multiple group setting is highly similar to the previous example, except that the factor correlation must now be provided for each group, so the Phi argument becomes a list containing the correlation in the first and second groups (of .00 and .20, respectively). Furthermore, any type of power analysis now expects the N argument, which gives the group weights in the case of an a priori power analysis. For instance, N = list(1, 1) specifies that both groups are of equal size. Given that cross-group comparisons of factor correlations are only meaningful when both loadings and factor variances are equal across groups, semPower 2 implements these invariance constraints when determining power and therefore requires that the measurement model is defined only once (which then applies equally to all groups). 
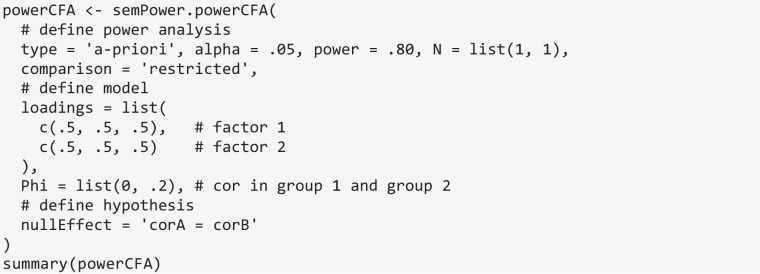


Running the power analysis indicates that *N* = 3116 observations (1558 per group) are required to detect that the specified factor correlation differs across groups with a power of 80%. Note again that this power estimate is only valid for this particular measurement model and the particular values assumed for the correlations. For instance, when replacing Phi above by Phi = list(.6, .8)*,* the required sample size is only* N* = 2286, although the difference between the correlations across groups remains .20.

semPower.powerCFA also supports hypotheses regarding the equality of correlations in a single-group design using nullEffect = 'corX = corZ'. Consider a CFA model involving two factors measured by three indicators each and an additional observed covariate. The factors are assumed to correlate by .50, and the correlations of the factors to the observed covariate are assumed to be .20 and .30, respectively. Phi now becomes a correlation matrix, where the first two columns refer to the factors, and the final column refers to the observed covariate. The loadings argument defines the loadings on the first factor to equal .5, .6, and .7, and those on the second factor to equal .8, .4, and .8, and defines the observed covariate by specifying a single loading of 1. Note that if a single loading equal to 1 is provided for all factors, semPower.powerCFA reduces to the special case of power analyses for correlation coefficients between observed variables (Olkin & Finn, 1995). Suppose the interest lies in detecting that the two factors correlate differently to the observed covariate. The nullWhich argument is a list comprising two vectors, jointly defining which elements in Phi to set to equality, so in the present example nullWhich = list(c(1, 3), c(2, 3)) targets the correlation between the first factor and the covariate as well as the correlation between the second factor and the covariate. Based on these input values, the required sample size is *N* = 1329.
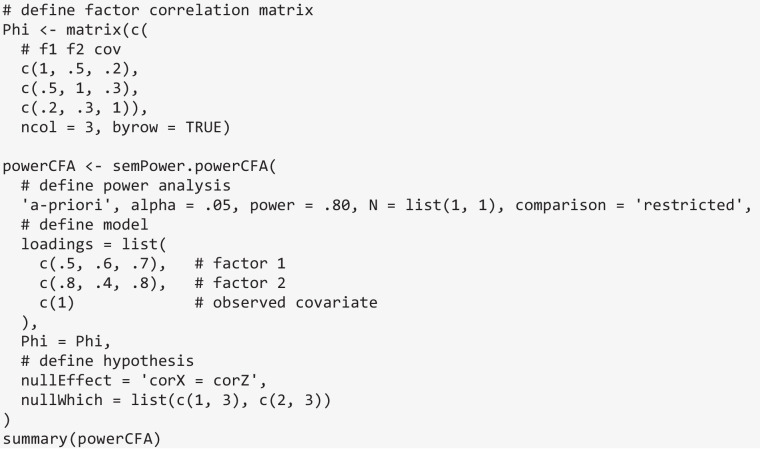


### Power analysis for hypotheses arising in a CLPM

The semPower.powerCLPM function can be used to perform power analyses concerning different hypotheses arising in a CLPM. In the present example, the CLPM involves two factors X and Y, each of which are measured at three waves. This allows for various relevant hypotheses: (a) detect that an autoregressive effect differs from zero, (b) detect that a cross-lagged effect differs from zero, (c) detect that the autoregressive effect of X differs from the autoregressive effect of Y, (d) detect that the cross-lagged effect of X differs from the cross-lagged effect of Y, (f) detect that an autoregressive effect differs from wave to wave, (g) detect that a cross-lagged effect differs from wave to wave, (h) detect that the synchronous residual correlations between X and Y differ across waves, (i) detect that an autoregressive effect differs across groups, and (j) detect that a cross-lagged effect differs across groups. In the following examples, we exemplarily consider power analyses to detect that a cross-lagged effect differs from zero, that the cross-lagged effect of X differs from the one of Y, and that the cross-lagged effect of X varies across waves.

While there are different ways for specifying a CLPM with three waves (nWaves = 3) (e.g., Newsom, 2015), a common approach is to place equality constraints on the autoregressive and cross-lagged effects on waves, so that there is only one autoregressive and one cross-lagged effect each for X and Y. This specification can be achieved by setting the waveEqual argument accordingly (waveEqual = c('autoregX', 'autoregY', 'crossedX', 'crossedY')). These equality constraints then apply to both the H0 model implementing the hypothesized effect and the H1 model omitting the restriction associated with the hypothesized effect. Similarly, when X and Y are latent factors (as in the present example), metric invariance over waves and autocorrelated indicator residuals are usually assumed. This is implemented by setting the arguments metricInvariance = TRUE and autocorResiduals = TRUE, which also applies to both models. The population values for the autoregressive effects for X and Y are provided in the autoregEffects argument, and take values of .60 for X and .70 for Y (both equal across waves). The cross-lagged effects (equal across waves) are defined in the crossedEffects argument, specifying a cross-lagged effect of X on Y of .10 and a cross-lagged effect of Y on X of .20. The synchronous (residual) correlations between X and Y are defined to be .30, .20, and .10 at the first, second, and third waves, respectively (rXY = c(.3, .1, .1)). Because the standardized argument is set to TRUE, all coefficients are treated as completely standardized, which implies that semPower 2 generates the (residual) variance–covariance matrix of the factors (${\boldsymbol{\Psi}}$) and the variance–covariance matrix of the indicators (${\boldsymbol{\Theta}}$) such that all variances are equal to 1. If standardized = FALSE, semPower 2 sets all diagonal elements of ${\boldsymbol{\Psi}}$ equal to 1, thus leading to unstandardized synchronous correlations, autoregressive, and cross-lagged effects. Finally, the hypothesis of interest is defined in the nullEffect argument. Here, the required sample size is requested to detect that the cross-lagged effect of X on Y (nullEffect = 'crossedX = 0') is ≥ .10 with a power of 80% on alpha = .05, which yields *N* = 958.
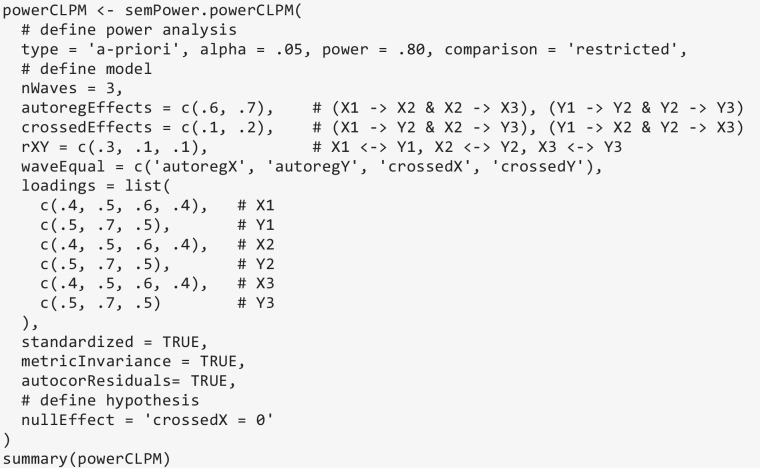


If the required sample size to detect that the cross-lagged effect of X (of .10) differs from the cross-lagged effect of Y (of .20) is to be determined, the only change refers to the nullEffect argument. Setting nullEffect = 'crossedX = crossedY' and rerunning semPower.powerCLPM gives a required sample of *N* = 2208 for a power of 80% on alpha = .05.

If the CLPM is based on observed variables (rather than latent factors), the measurement model needs to be defined such that there is a single loading of 1 for each factor, which is most easily done by providing an identity matrix to the Lambda argument. For instance, when replacing the loadings argument by Lambda = diag(6) in the previous example, the required sample size to detect that the cross-lagged effects of X and Y differ with a power of 80% is *N* = 449. The metricInvariance and autocorResiduals arguments are ignored for the observed variables.

The previous examples implemented the CLPM assuming constant autoregressive and cross-lagged effects across waves. Wave-dependent effects can be specified by dropping the respective entries in the waveEqual argument and providing lists to the autoregEffects and crossedEffects arguments. In the following example, the autoregressive effects are still constant across waves, whereas the cross-lagged effect of X on Y is .10 from wave 1 to 2, but .30 from wave 2 to 3. Then, the required sample size to detect that the autoregressive effects of X vary across waves (nullEffect = crossedX) is requested, which yields *N* = 1348.
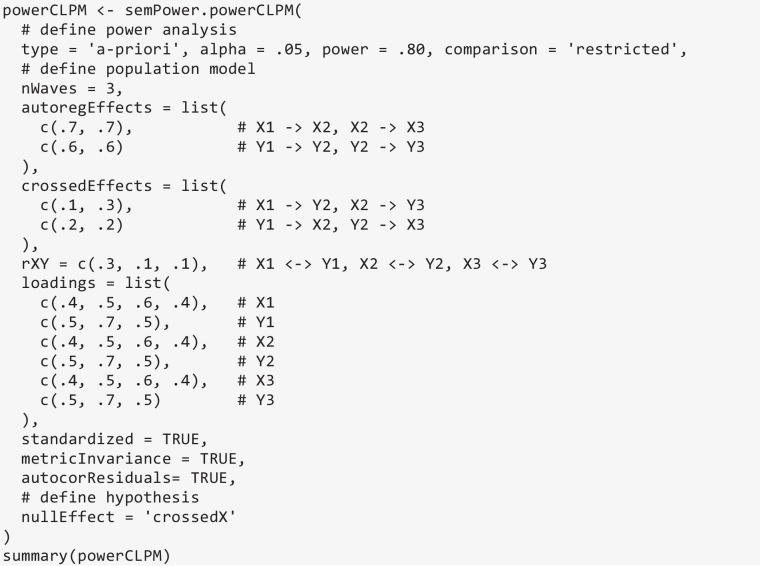


### Power analysis for hypotheses arising in a generic path model

Although semPower 2 contains several functions for special regression problems (such as a single linear regression, mediation structures, and CLPMs; see Table 1), some regression-related hypotheses are not covered. Therefore, semPower 2 also provides a more generic function to perform model-based power analyses arising in path models that requires specification of **B** and, optionally, ${\boldsymbol{\Psi}}$.

As an example, consider a model involving two correlated factors and two correlated observed outcomes. A substantive hypothesis might be that the slope of the first factor in the prediction of the first outcome does not differ from the slope of the second factor in the prediction of the second outcome. The Beta matrix defined in the code below sets up the regression relationships in the population and implies a slope for the first and second factors of .20 and .10, respectively, in the prediction of the first outcome, and a slope for the first and second factors of .10 and .40, respectively, in the prediction of the second outcome. Further, the Psi matrix defines a correlation between the factors of .30, and a residual correlation between the outcomes of .20. If the standardized argument is set to TRUE, all slopes are interpreted in a standardized metric and the matrix provided to Psi is interpreted as the matrix of (residual-)correlations, so that the actual ${\boldsymbol{\Psi}}$ is computed such that all variances are equal to 1. If standardized = FALSE, the coefficients in Beta and Psi are directly used to define **B** and ${\boldsymbol{\Psi}}$, thus (in general) implying unstandardized coefficients. Finally, the hypothesis that the slope of the first factor in the prediction of the first outcome is equal to the slope of the second factor in the prediction of the second outcome is defined in nullEffect = 'betaX = betaZ' along with the nullWhich argument, which is a list containing vectors referring to which elements in Beta to constrain to equality. Running semPower.powerPath with these arguments leads to a required sample size of *N* = 840 to detect the specified effect with a power of 80%.
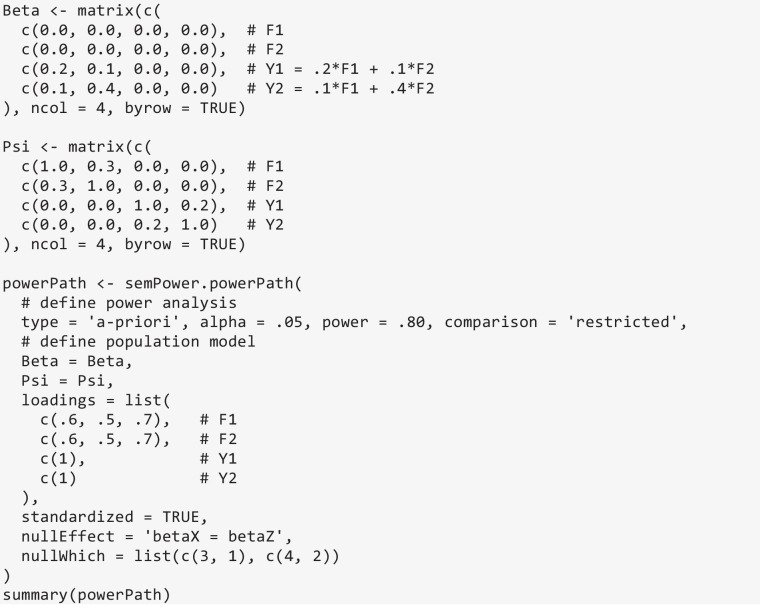


### Generic model-based power analysis

semPower 2 also provides a generic function to perform model-based power analyses for scenarios not covered in any more specific function. Consider the situation in which one is interested in determining whether the observed responses on eight indicators reflect two separate (but correlated) factors or can be described by assuming just a single factor, and that a correlation of *r* ≥ .90 is considered to imply that a single factor is sufficient. A suitable H0 model to test this hypothesis would specify two factors and constrain their correlation to 1. Although the model conforms to a standard CFA model, this particular hypothesis is not covered by the semPower.powerCFA function, so the more general semPower.powerLav function needs to be employed.

semPower.powerLav requires a lavaan model string defining the H0 model (and optionally a model string defining the H1 model), and either a lavaan model string defining the population model or a population variance–covariance matrix (and mean vector, if necessary). A useful utility function supporting the definition of the population variance–covariance matrix is semPower.genSigma, which expects the model matrices (see Eqs. 6-9) as input, but accepts the same shortcuts as any other function performing model-based power analysis. For instance, the following generates the implied covariance matrix from a population model involving two factors that are correlated by .90 and are measured by four indicators each.
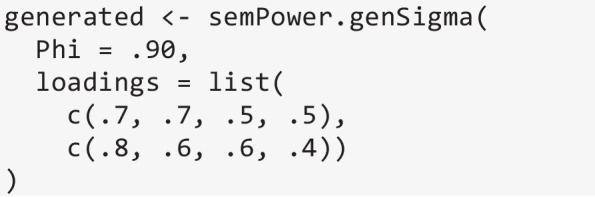


Beyond the implied covariance matrix (Sigma), the results variable returned by semPower.genSigma also includes a lavaan model string (modelTrue) created such that the model perfectly describes the population. In the present example, this is a simple two-factor model that freely estimates the factor correlation, and thereby represents a suitable H1 model. The model string representing the H0 must restrict the factor correlation to be equal to 1:
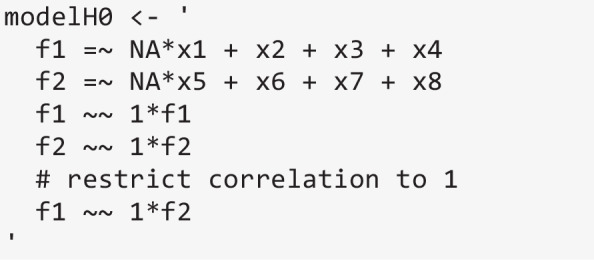


Finally, everything is plugged into semPower.powerLav, in the present example requesting an a priori power analysis, which shows that 323 observations are required to detect a factor correlation < .90 with a power of 80%.
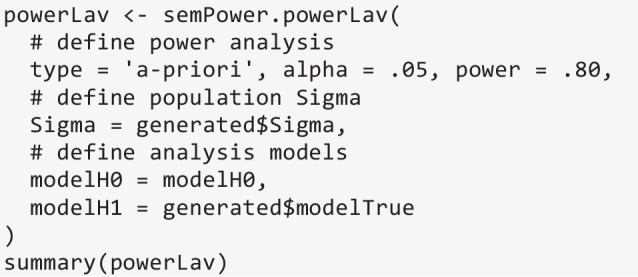


### Simulated power analysis

All model-based power analyses provided by semPower 2 can also be performed as a simulated, rather than analytical, power analysis by adding the argument simulatedPower = TRUE to the respective function call. In addition, a list with options controlling the simulation process can be provided via the simOptions argument, allowing for changing the number of replications, parallel processing, and the generation of non-normal data and/or missingness. For instance, the following code performs a simulated a priori power analysis concerning the correlation in a two-factor model using the default settings (i.e., 500 replications, multivariate normal and complete data, ML estimation), and additionally requests parallel processing using four cores (simOptions = list(nCores = 4)):
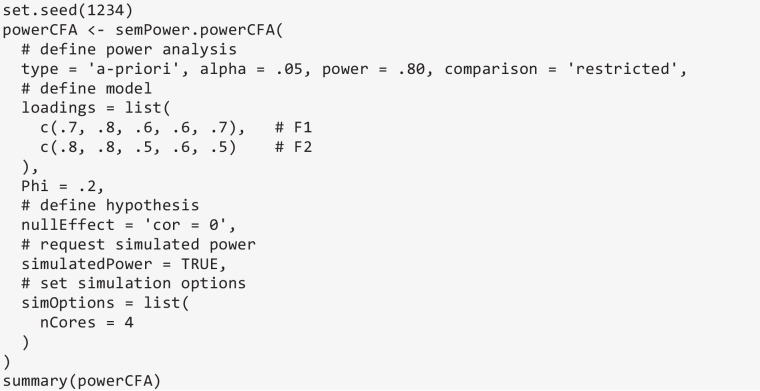


Given that simulated power analyses are arguably most useful when the data generation process implements violations of the assumptions underlying the chosen estimator (and as a consequence also the assumptions underlying analytical power analysis), semPower 2 includes options to generate non-normal data and data with missing values. Generating multivariate non-normal data with known covariance structure is an intricate issue and active area of research. Therefore, semPower 2 interfaces four different data generation methods, namely Foldnes and Olsson (2016), Qu et al. (2020), Ruscio and Kaczetow (2008), and Vale and Maurelli (1983), each of which is associated with different multivariate distributions that may affect the empirically observed χ^2^-distributions differently (e.g., Auerswald & Moshagen, 2015; Foldnes & Grønneberg, 2015; Jobst et al., 2022) and requires different input. As an example, the following code uses the Vale-Maurelli approach (type = 'VM') specifying symmetric (skewness), but moderately to strongly kurtotic (kurtosis) marginal distributions. Moreover, there are also missing data on the first and second indicator of the first factor as well as the final two indicators of the second factor (missingVars = c(1, 2, 9, 10)), generated based on a missing-at-random mechanism (missingMechanism = 'MAR') with a missing proportion of 25% (missingProp = .25). For MAR, semPower 2 implements the percentile method generating the maximum number of missing patterns (Zhang, 2021). When missing data are generated, semPower 2 resorts to full information maximum likelihood estimation.
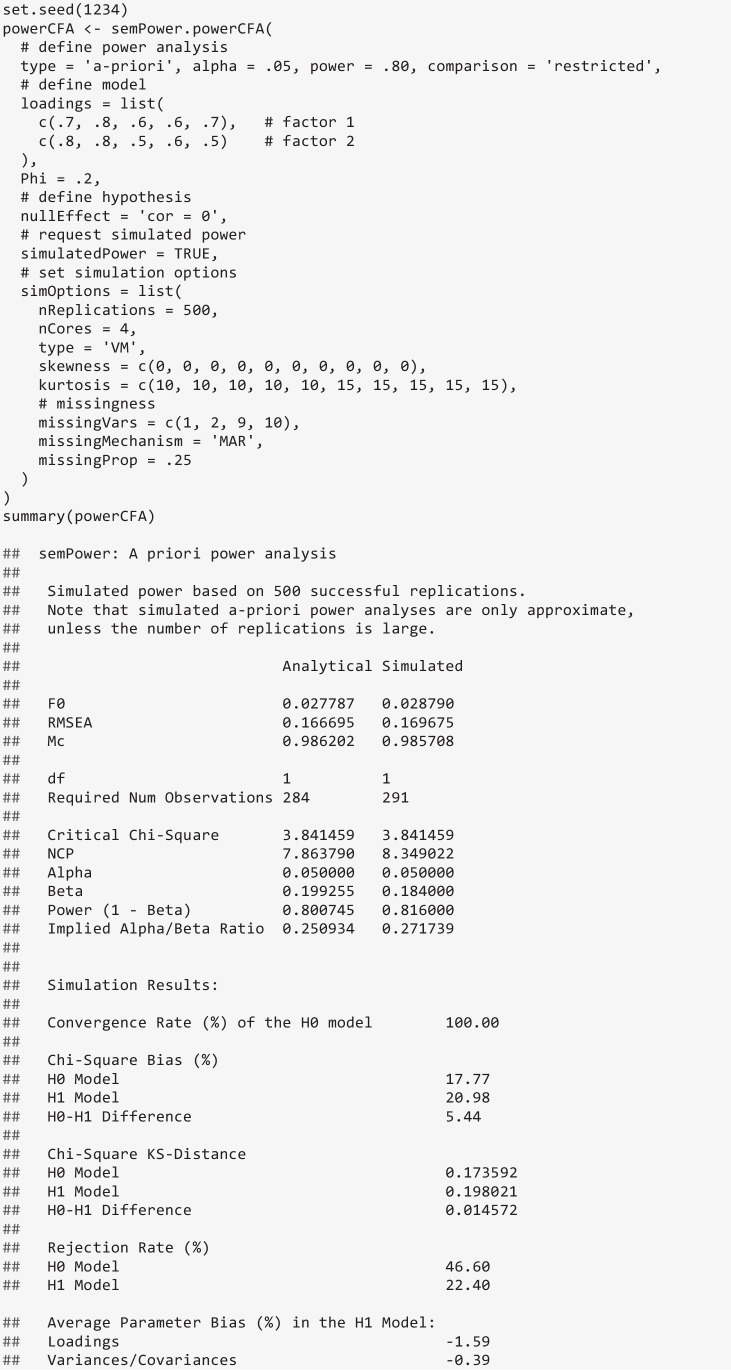


The output now contains an additional column containing the simulation results. Note that the measures of effect refer to the estimates of the population values (rather than being averaged sample values), so these can be immediately compared across the different approaches to power analysis. Of note (and perhaps surprisingly, but consistent with Steiger et al., 1985), the simulation results very closely match the analytical results in terms of the sample size required to detect the hypothesized effect despite the non-normal and incomplete data.

The output further reports on the simulation results in greater detail. In particular, the convergence rate (of the H0 model) and the average relative parameter bias by type of model parameter is provided (i.e., $RB = 100 \cdot (\widehat{\theta }-\theta )/\theta$), jointly giving an indication of whether the sample size is sufficient to support successful parameter estimation of the model under scrutiny. In addition, the empirically observed central and noncentral χ^2^ distributions are compared against the respective asymptotically expected reference distribution using three measures. First, the relative bias evaluates the departure of the empirical mean from the expected value ($RB = 100 \cdot (\overline{{\upchi }^{2}}-df)/df$ and $RB = 100 \cdot (\overline{{\upchi }^{2}}-(df +\uplambda ))/(df +\uplambda$)), respectively). Second, the average Kolmogorov–Smirnov (KS) distance measures the discrepancy between the empirical and the reference cumulative distribution functions akin to the Kolmogorov–Smirnov test statistic, but relies on the average, rather than the maximum, absolute distance, $KS = M(|{F}_{e}(x) - {F}_{r}(x)|$) (see Yuan et al., 2007). The average KS distance thus ranges from 0 (for no difference) to .50 (for completely nonoverlapping distributions). Finally, the empirical rejection rate given the specified alpha-error is provided as a measure of how strongly the tail of the empirical distribution differs from the asymptotic reference distribution. When the model representing the H0 is compared against the saturated model, this is the same as the empirical power estimate.

In the example above, the relative bias and the KS-distance indicate strong departures of the empirical distributions from the respective asymptotic distribution, which is also mirrored by a rejection rate of the (correctly specified) H1 model that clearly exceeds the nominal alpha-error of 5%. However, the difference distribution closely follows the theoretically expected distribution, so there is virtually no difference (beyond sampling error) between the analytical and the simulated power estimate. Indeed, it is our experience that simulated power analyses for the comparison against an explicit H1 model usually show only minor departures from corresponding analytical power analyses. When the comparison is performed against the saturated model, however, simulated power is often higher as a consequence of the well-documented positive bias of the model test when the assumptions are violated (e.g., Curran et al., 1996; Moshagen, 2012).

By default, semPower 2 performs simulated power analyses employing ML estimation with the uncorrected model test statistic. However, semPower 2 also allows one to change the estimator and test statistic employed in a simulated power analysis by setting the lavOptions argument, which is passed to lavaan and thus conforms to the standard lavaan conventions. For instance, lavOptions = list(estimator = 'mlr') leads to a simulated power estimate relying on a corrected test statistic that is asymptotically equivalent to the Yuan & Bentler (2000) statistic, whereas the analytic power estimate is still based on the asymptotically expected distributions based on ML. Repeating the previous example with this additional argument yields less biased distributions of the corrected test statistic under both the H0 and the H1 as compared to the results obtained above using the uncorrected test statistic, Note that the simulated power estimate is still very close to the analytical power estimate:
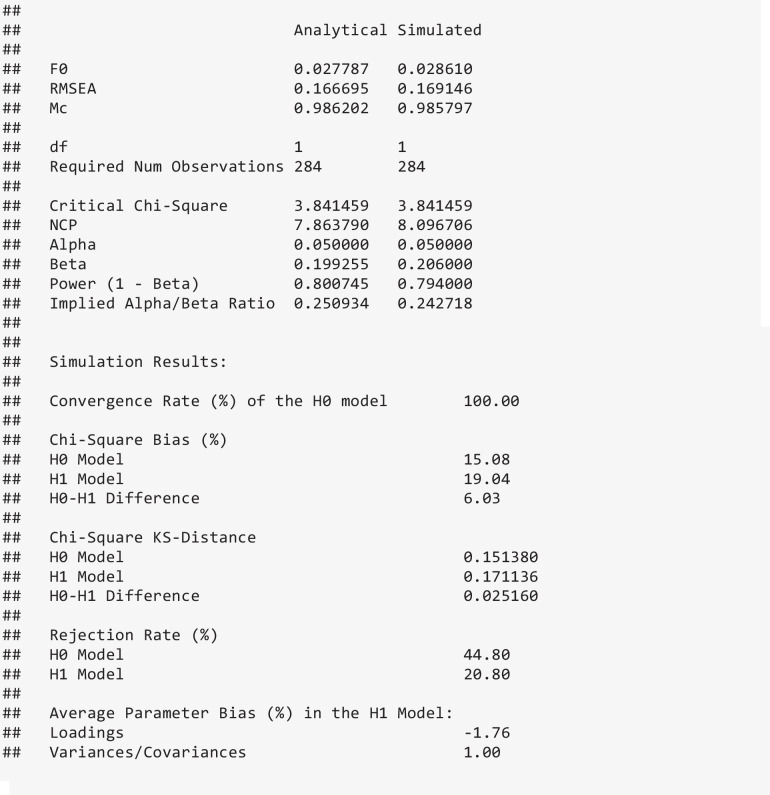


## Conclusion

The present paper provided an introductory overview of the semPower 2 package for the R environment, which provides both analytical and simulated a priori, post hoc, and compromise power analysis based on either a model-free or a model-based definition of the effect and provides high-level functions to simplify the definition of meaningful hypotheses arising in popular types of models (which are to be expanded to cover additional models in future versions). semPower 2 thus offers a high degree of both accessibility and flexibility in performing power analysis, and thereby overcomes several limitations of previous tools providing either analytical or simulated power analysis (but not both), thus complicating a direct comparison across these approaches. In addition, most existing tools offering simulated power analysis provide post hoc power analyses only, often consider particular model types, require the cumbersome and error-prone definition of the values of all population parameters, and do not offer easily accessible options to change the estimator or to embed non-normally distributed data and or different patterns of missingness in the simulation process.

Although the examples provided in the present paper focused on power analysis concerning models that include latent variables, semPower also provides power analysis of correlation and regression problems involving only observed variables as a special case. As such, we hope that semPower 2 may promote the use of power analysis in determining the required sample size and evaluating hypothesis tests in a meaningful way more generally.

Limitations of the current version of semPower 2 include that multilevel models and models involving ordered categorical data are not yet supported. Also, semPower 2 is operated entirely through the R language. Although a Shiny app providing model-free power analyses is available at https://sempower.shinyapps.io/sempower, there is currently no graphical user interface covering the complete functionality of semPower 2. Readers who are more comfortable with a graphical user interface are referred to Jak et al. (2021) for analytical and to Wang and Rhemtulla (2021) for simulated power analyses.

Finally, it must be stressed that the prime purpose of semPower 2 is to perform power analyses concerning a particular hypothesis. Although the simulation option provides some indication of whether the required sample size yielding a particular power is also sufficient to support successful estimation of the model parameters, the question of whether a certain sample size guarantees proper convergence and good parameter recovery is not the focus of semPower 2. Statistical power is only one of several aspects to consider in determining sample size requirements.

## Footnotes

1 The regularity conditions include the so-called parameter-drift assumption, which states that the degree of misspecification may not be too large relative to the degree of sampling error.

2 McDonald’s (1989) index of non-centrality is computed from the minimum of the fit function by $\mathrm{Mc }=\mathrm{ exp}(-.5\cdot {\mathrm{F}}_{0})$, so that the Mc ranges from 0 to 1, with higher values indicating better fit.
